# Sensory Profile-2 in Autism Spectrum Disorder: An Analysis within the International Classification of Functioning, Disability and Health Framework

**DOI:** 10.1007/s10803-024-06337-y

**Published:** 2024-04-13

**Authors:** Marta Marcilla-Jorda, Catarina Grande, Vera Coelho, César Rubio-Belmonte, Micaela Moro-Ipola

**Affiliations:** 1 Faculty of Health Sciences, Universitat Jaume I, Castello de la Plana, Spain; 2 Faculty of Psychology and Education Sciences, University of Porto, Porto, Portugal; 3 Department of Social and Behavioral Sciences, Universidade da Maia, Maia, Portugal; 4 Universidad Católica de Valencia San Vicente Mártir, Valencia, Spain; 5 Center for Psychology at University of Porto, University of Porto, Porto, Portugal

**Keywords:** International Classification of Functioning Disability and Health Children and Youth Version (ICF-CY), Autism Spectrum Disorder (ASD), The Sensory Profile™ 2 (SP-2), ICF-Core Sets

## Abstract

Autism spectrum disorder (ASD) is characterized by impairments in many functional areas requiring long-term interventions to promote autonomy. This study aims to map The Sensory Profile™ 2 (SP-2), one of the most widely used assessment tools in children with ASD, with the International Classification of Functioning, Disability and Health for Children and Youth (ICF-CY), developed by the World Health Organization (WHO). This will allow the identification of the functional dimensions covered by this instrument and the comparison with the ICF shortlist proposed for autism (ICF Core Set [ICF-CS]). The deductive content analysis described in the ICF Linking Rules was followed, along with a systematized process including statistical and reasoning techniques that could contribute to the improvement of ICF linking studies (Cohen’s Kappa and percentage of agreement). 218 codes were identified, 71% of them were codes related to the body functions chapters, mainly linked to perceptual functions (b160), emotional functions (b152), and temperament and personality functions (b126). Concerning activities and participation chapters (29%) the most frequently used codes were: focusing attention (d160), carrying out daily routine (d230), and walking (d450). Even though the SP-2 items do not assess most of the functional features regarded as essential in the ASD ICF-CS, SP-2 encompasses a majority of problems concerning body functions. This instrument may be considered as part of a multidimensional assessment approach, to complement other sources that are more likely to assess activity and participation dimensions and guide a functional intervention.

## Introduction

The worldwide prevalence of autism spectrum disorder (ASD) has increased over the last decades (Zeidan et al., 2022). The Autism and Developmental Disabilities Monitoring Network estimates one in 36 the prevalence of ASD among children aged 8 in the United States (Maenner et al., 2023). Under the fifth edition of the Diagnostic and Statistical Manual of Mental Health Disorders (DSM-5) criteria, ASD is a neuro-developmental disorder characterized by impairments in communication and social interaction, combined with highly restricted interests and/or sensory behaviors (DSM-5, American Psychiatric Association [APA], 2014). ASD have their onset in early childhood but is persistent along its lifetime and frequently requires intensive long-term support and educational strategies because of the impact of these traits in many functional areas as daily living activities (such as bathing or dressing) (Duncan & Bishop, 2015; Jasmin et al., 2009; Travers, 2017), instrumental activities of daily living (as running errands or shopping) (Baker et al., 2023; Fortuna et al., 2016), social skills (Howlin et al., 2013), work (Ezerins et al., 2023), education (Eldar et al., 2010) or sleep (Estes et al., 2023; Park et al., 2012; Schiltz et al., 2022).

**Fig. 1:**
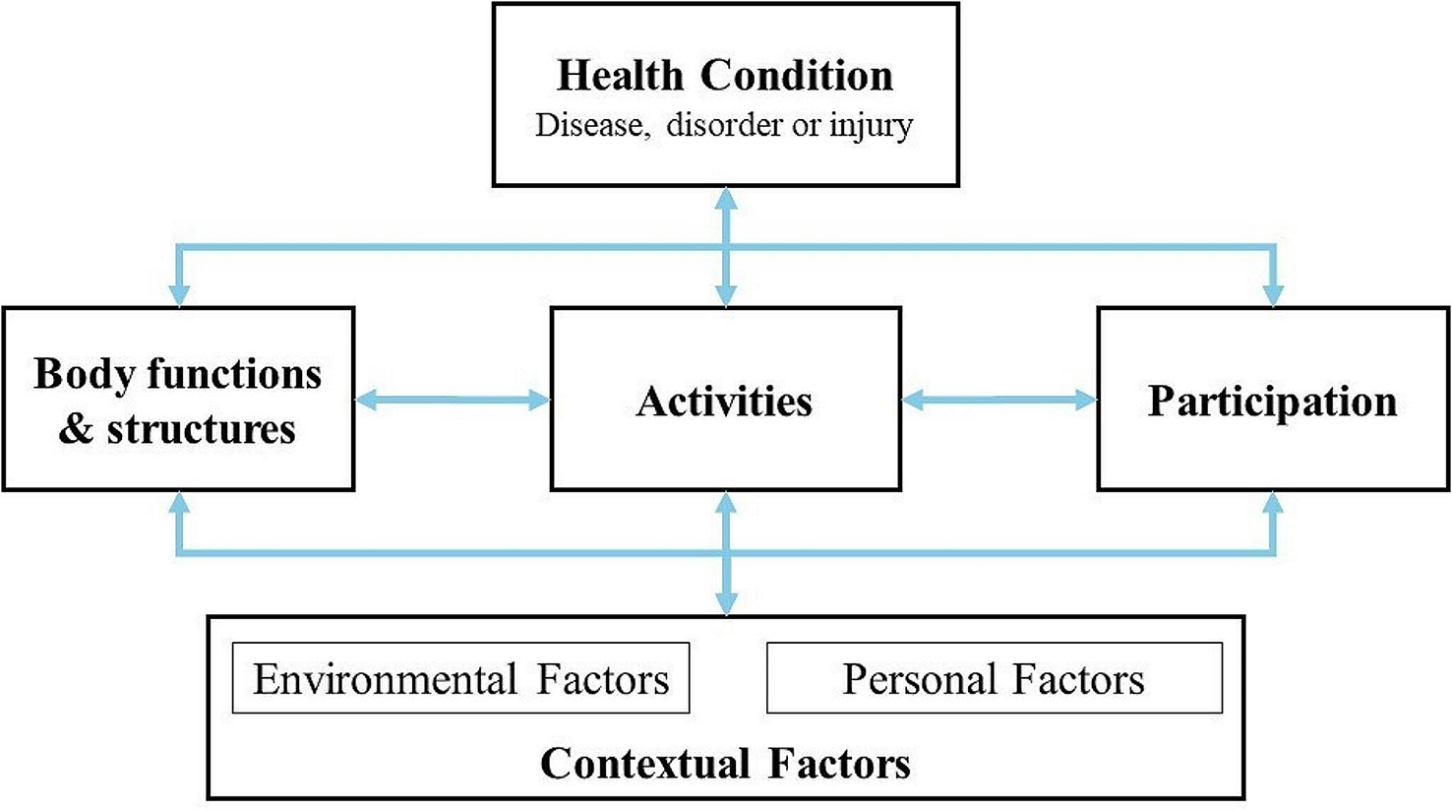
Interactions between the components of International Classification of Functioning, Disability and Health, adapted from the ICF (WHO, 2001)

The importance of addressing these aspects has been especially highlighted by the World Health Organization (WHO), which considers full participation in daily living activities and routines as a main component of health (WHO, 2001). It is under this perspective that the International Classification of Functioning, Disability and Health (ICF), (WHO, 2001) and its Children and Youth version (ICF-CY) (WHO, 2007) were developed. ICF is a recommended framework for assessment and intervention planning, both for health and human service professions (Castro & Palikara, 2017; Simeonsson, 2009). Under the ICF framework, functioning is described considering three components: body systems (functions and structures), activities and participation, and the influence of contextual factors (environmental and personal factors). ICF and ICF-CY describe states of functioning and health with an alphanumeric coding system. The code starts with a letter to discern between components: “b” for Body Function, “s” for Body Structures, “d” for Activities/Participation and “e” for Environmental Factors (see Fig. 1). This letter is followed by a numeric code that starts with the chapter number (one digit), followed by the second level heading (two digits), and the third and fourth level headings (one digit each). The more levels are described, more precisely the information shared by the code is (WHO, 2001, 2007). For example, the code *d3100 “Responding to human voice”*, is from the component *“activities and participation (d)”*, chapter 3 *“Communication (d3)”* and part of the second level heading “*Communicating with - receiving - spoken messages (d310)”.*

ICF Core Sets (ICF-CS) were developed for a comprehensive, yet efficient application of the ICF in daily clinical practice and research (Selb et al., 2015; Yen et al., 2014). ICF-CS are shortlists of ICF categories selected from the full ICF classification that are considered necessary to describe the functioning of a person with a specific health condition (Bickenbach et al., 2012). The Comprehensive ICF-CS for ASD, used in the present study, includes 111 second level International Classification of Functioning, Disability and Health categories: one body structure (1%), 20 body functions (18%), 59 activities and participation (53%), and 31 environmental factors (28%) categories (Bölte et al., 2019; Schiariti et al., 2018). There is also a Common Brief ICF-CS with 60 categories and age-appropriate ICF-CS: (a) the preschool version (0- to 5-year-old children), (b) the school-age version (6- to 16-year-old children and adolescents), and (c) the older adolescent and adult version (⩾17-year-old individuals) with 73, 81, and 79 categories, respectively (Bölte et al., 2019). The ICF-CS is an important tool for improving mutual understanding and communication between individuals with chronic health conditions, their families and health professionals regarding their functioning and intervention goals (Fernández-López et al., 2009).

One of the clinical applications of the ICF is the linking process of the most commonly used instruments in every health field to ICF categories, and also to specific ICF-CS relevant to neuro-developmental conditions as ASD (D’Arcy et al., 2022; Hayden-Evans et al., 2022). Previous studies have shown the usefulness of this linking process in analyzing measurements used for diagnosing children with ASD such as the Autism Diagnostic Observation Schedule (ADOS-2, Lord et al., 1989) and the Autism Diagnostic Interview (ADI-R, Rutter et al., 2003a) (Castro et al., 2013; Black et al., 2023) as well as the Modified Checklist for Autism in Toddlers (M-CHAT, Robins et al., 2001), the Social Communication Questionnaire (SCQ, Rutter et al., 2003b) the Childhood Autism Rating Scale (CARS, Schopler et al., 2010) (Black et al., 2023) and typical measures of functioning used in the assessment of ASD such as the ABAS-3 (Harrison & Oakland, 2015) or the VINELAND-3 (Sparrow et al., 2005) (Hayden-Evans et al., 2022). This process enables researchers to identify and compare the meaningful units included in different assessment tools with the ICF and provides information about the functional aspects covered by a specific instrument for a health condition (Fayed et al., 2012). Moreover, it may lead to an individualized intervention planning and to adopt a functional approach in the assessment-intervention process.

Identifying the presence of sensory processing dysfunction has become a relevant aspect to assess in individuals with ASD since the recent incorporation of sensory difficulties as part of the ASD diagnosis (DSM5, APA, 2014). From the ICF approach, it also has a relevant implication because of the association between sensory abnormalities and poor functional outcomes, behavioral difficulties, and autism severity across the lifespan (DuBois et al., 2017). The Sensory Profile™ 2 (SP-2) (Dunn, 2014) is one of the most used assessment tools in this matter (Burns et al., 2017).

The main goal of this study is to generate a construct analysis of the SP-2 content using the ICF-CY framework. Related to this, three specific objectives were also formulated:

- To study the agreement level between two ICF-CY trained researchers along the linking process between the ICF-CY functioning dimensions and the SP-2.
- To describe the distribution of the SP-2 items after being linked to ICF-CY codes within the body functions, activity and participation and environmental factors components.
- To identify the functional dimensions considered essential from the ASD ICF-CS that are assessed by SP-2.

## Methods

### Instrument

The Sensory Profile™ 2 (Dunn, 2014) in its child version (from 3:0 to 14:11 years old) for caregivers, assesses the child’s response to sensory events throughout daily living situations. It comprises 86 items which are answered in a 6-point scale scored: Always or almost always (5), Frequently (4), Half of the times (3), Occasionally (2), Seldom or never (1), and Not applicable (0).

This instrument is based on Dunn’s Sensory Processing Model (1997) which theorizes the relationship between: (a) the functioning of a person’s nervous system (neurological construct) and (b) self-regulatory strategies (behavioral construct). In terms of sensory input processing (neurological construct), this model states that each person has a personal range of thresholds (different for each type of sensory information) for noticing and responding to sensory events in everyday life. Thus, a low sensory threshold means that the system is easily activated and therefore will notice and respond to stimuli quite frequently; while a high threshold means that the system requires stronger stimuli to activate and therefore will miss stimuli that are easily noticed by others. In terms of responding to sensory stimuli (behavioral construct), on a continuum, people let things happen around them and then react (passive strategy) or people tend to do things to control the amount and type of input available to them (active strategy).

The interaction of these neurological and behavioral constructs creates 4 basic patterns of sensory processing: (a) sensation seeking (high thresholds and an active self-regulation strategy); (b) sensation avoiding (low thresholds and an active self-regulation strategy); (c) sensory sensitivity (low thresholds and a passive self-regulation strategy); and (d) low registration (high thresholds and a passive self-regulation strategy). These four different subtypes of sensory responses are analyzed by the SP-2 in 9 sensory processing sections: Auditory processing, Visual processing, Touch processing, Movement processing, Body Position Processing, Oral Processing, Conductual response, Socio-emotional response, and Attentional response.

The SP-2 has been widely used in earlier studies, including the population with ASD (DeBoth & Reynolds, 2017; Kientz & Dunn, 1997; McConachie et al., 2015; Simpson et al., 2019; Tomchek & Dunn, 2007; Watling et al., 2001). The reliability and validity have been extensively studied with good results (Brown et al., 2001, 2008; Dean et al., 2016; Dean & Dunn, 2018). The SP-2 has been translated into different languages, including Spanish, the version used during the linking procedure.

### Linking the Sensory Profile™ 2 and the ICF-CY

To achieve the purpose of the study, each item of the SP-2 was linked with the ICF-CY classification system. Linking process followed the deductive content analysis of the published Linking Rules (Cieza et al., 2002, 2005, 2016) developed for the specific purpose of connecting assessment tool content with the ICF. The linking process and resulting analysis were carried out by three psychologists and two occupational therapists. All researchers were familiar with the assessment tool, Dunn’s Model of Sensory Processing and had solid knowledge and training on the use of the ICF-CY system and the ICF linking rules.

**Fig. 2:**
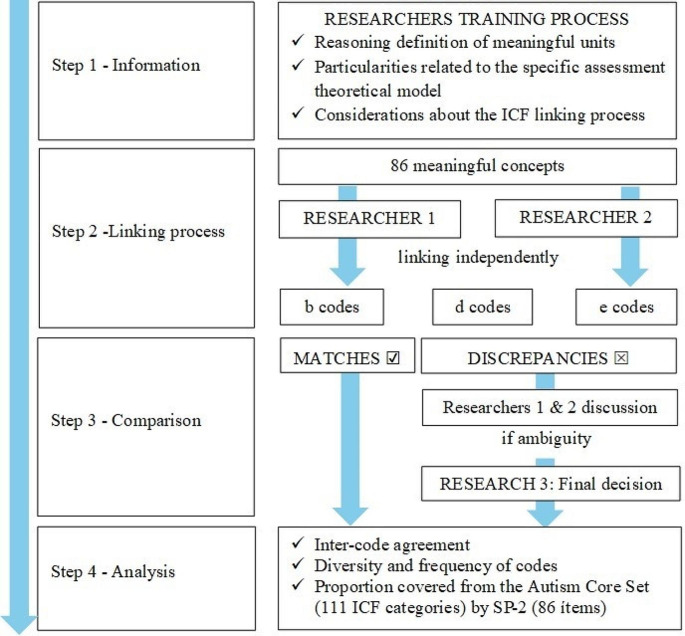
Linking ICF-CY with SP-2: Linking process flow chart

Four steps were followed in the present study (see Fig. 2):

### Step 1: Information

As it is outlined in the linking rules, performing the linking process requires a good understanding of the concepts, definitions, and structure of the ICF/ICF-CY (Cieza et al., 2016). In order to achieve this common knowledge, the researchers went through a training process where they agreed on the coding criteria (Fayed et al., 2012). As recommended by Cieza et al., 2016, all the steps of this process were documented in a research diary that included:

- The reasoning behind the definition of the different meaningful units: In a questionnaire (or a test like SP-2) concepts are identified within items, but developing a linking ICF process requires transforming these items into meaningful units. A meaningful unit is a specific unit of text (from a few words to a sentence) that maintains a common theme (Karlsson, 1993) and does not follow linguistic grammatical rules (Stucki et al., 2008). This way, transforming the items into meaningful units, allows to extract the relevant information of the item in order to link them to an exact ICF code. For example, if the researchers had to identify the meaningful units in this item “*Is distracted or has trouble functioning if there is a lot of noise around, like the radio or the tv*” the researchers established that the meaningful unit is the ability to sustain attention in a noisy environment, thus this will be the concept coded afterwards.

Also, the decision was made not to integrate the examples of the item as part of the meaningful unit.

b) Particularities related to the specific assessment theoretical model that could influence the linking process: For example, the consideration of The SP-2 assessed in every item (seeker, avoiding, sensitive, or registration) to be properly linked to the ICF-CY code.
c) Considerations about the ICF linking process, such as:

- Establishing that only the behaviors required to meet the item specifications would be coded, and not the other behaviors involved in the activity. To exemplify this situation, if the researchers had to identify the meaningful units in the item *“Doesn’t seem to notice when face or hands are messy”*, the information relating to the child’s ability to apply water, soap and other substances to body parts such as hands to clean them (d5100 washing body parts) were not considered, as the assessment purpose of this item is related to the touch processing. In order to be able to assign the most precise code to each meaningful unit, following the item’s purpose of assessment, the whole item should be read before splitting it into a meaningful unit.
- Accepting that, if a meaningful unit could be linked to two different ICF categories, both codes would be used, making sure that the true purpose of the item was reflected.
- Avoiding “Other Specified” or “Unspecified” codes whenever possible.

### Step 2: Linking Process

Secondly, after identifying 86 meaningful units from the 86 SP-2 items, two researchers independently went through the linking process.

The coding procedure followed similar steps to previous studies in which a similar mapping process was conducted between assessment measurements and the ICF-CY (Black et al., 2023; Castro et al., 2013, 2016; Castro & Grande, 2018; Dahlgren et al., 2013; Sogo, 2020). Every meaningful unit was linked to a body function (b), activities and participation (d) or environmental factor (e) code and entered into a spreadsheet for its subsequent comparison and analysis.

### Step 3: Comparison

Coding results were compared to detect matches and discrepancies. To reach a final coding agreement, discrepancies were discussed until an agreement was reached. In the case of ambiguity, a third researcher was consulted to make a final decision on the most appropriate linking.

### Step 4: Analysis

During this phase, 3 different analyses were developed:

- *The calculation of the inter-coder agreement*: This includes the Kappa correlation coefficient (Cohen’s Kappa) and the percentage of agreement between the two raters in each item (PA).

The Kappa correlation coefficient is a reliability index for the proportion of agreement between coders in nominal scales. Kappa values vary between − 1 (total disagreement) and 1 (perfect agreement); a value of zero means that the agreement found is due to chance. Landis and Koch (1977) describe the strength of the agreement of Kappa as: 0–0.20 slight, 0.21–0.40 fair, 0.41–0.60 moderate, 0.61–0.80 substantial, and 0.81–1 almost perfect. Kappa was calculated for each ICF-CY code that was assigned to any concept within each analyzed item. For example, code b1141 was used by the first coder to refer to only one item, and not used in the other 85 items. The second coder happened to be the same, so the Kappa obtained for that code was 1. Cohen’s Kappa was calculated with an online calculator from Idostatistics (https://idostatistics.com/cohen-kappa-free-calculator/). Mean Kappa values were calculated twice: assuming every code was used just once and including the actual frequency of every code.

The percentage of agreement between the two raters in each item (PA) was obtained following the proposal of Østensjø et al. (2006). The linking process for every item was classified as: full agreement, partial agreement, or non-agreement. Total agreement meant that in an item both coders agree at least on one exact code (fourth level headings of the ICF-CY classification). Partial agreement means that, in one item, coders agree at least in a third level of the ICF classification. It was considered No agreement for the rest of the cases. For instance, if the first coder linked an item to d1600 (Focusing attention on the human touch, face and voice) but the second coder believed it should be linked to d1601 (Focusing attention to changes in the environment) the PA analysis would note this as a partial agreement, as both coder had agreed on the ICF component, chapter and activity but not on the exact four-level code. In another example if the first coder considers an item to be linked to d160 (Focusing attention) and the second coder considers it to be linked to d161 (Directing attention), even though they agreed on the same component and chapter, it is considered as no agreement.

- *Diversity and frequency of codes*: The study of the distribution of the SP-2 items codes across the ICF-CY chapters, analyzing the diversity and frequency of codes.
- *Proportion covered from the Autism Core Set by SP-2*: The comparison between the codes and the ASD ICF-CS (Bölte et al., 2019; Schiariti et al., 2018).

## Results

Between one and four codes were matched with every meaningful unit through the linking process. Most of the meaningful units needed two codes to cover all the aspects of the item (this happens in 35 of the 86 meaningful units). Table 1 describes every meaningful unit, associated ICF-CY code and Kappa agreement.

**Table 1:** Distribution of SP-2 meaningful units linked to the International Classification of Functioning, Disability and Health components and chapters and Kappa correlation

SP-2 Section^a^	SP-2 Subtype	SP-2 Item	Meaningful item	Final coding			
				Body functions		Activity and participation	
				ICF-CY Code	Kappa	ICF-CY Code	Kappa
A	EV	1	Intense emotional/behavioral response to loud or unexpected noises	b1520 b1560 b2703	0.91 1 0.69	NONE	-
A	EV	2	Hold hands over the ears to avoid sound	b1560 b2703 b755	1 0.69 0.39	NONE	-
A	SE	3	Difficulties to complete tasks in environments with background sounds	b1560	1	d210	1
A	SE	4	Sustaining attention in noisy environments	b1400 b1560	0.85 1	d160	0.90
A	EV	5	Completing tasks in noisy environments	b1560	1	d210	1
A	SE	6	Difficulties related to respond to orders or human voice	b1560	1	d1600 d3100	0.85 1
A	SE	7	Absence of response to his/her own name	b1560	1	d160 d3100	0.90 1
A	RE	8	Enjoys/positive emotions listening to noises /Makes sounds for pleasure	b1520 b1560 b3401	0.90 1 0	NONE	-
B	SE	9	Sensory preference to perform activities in dim light	b1301 b1561 b21020	0.75 0.71 0.85	NONE	-
B	NQ	10	Visual sensory preference for dressing with bright-colored or patterned clothes	b1301 b1561	0.75 0.71	NONE	-
B	NQ	11	Positive emotions related to look carefully or intensively at objects	b1520 b1561	0.91 0.71	NONE	-
B	RE	12	(Difficulties) finding objects	b1565	0	d1601	0
B	SE	13	Negative emotions related to bright light	b1520 b1561 b21020 b2703	0.91 0.71 0.85 0.69	NONE	-
B	BU	14	Stares at people moving (focusing)	b1561	0.71	d110	0.56
B	EV	15	Bright lights bother him/her	b2703 b1561 b21020	0.69 0.71 0.85	NONE	-
C	SE	16	Negative emotions related to be touched by someone else	b1520 b1564 b2702 b2703	0.91 1 0.39 0.69	NONE	-
C	NQ	17	Negative emotions in response to wearing shoes or socks	b1520 b1564 b2702 b2703	0.91 1 0.39 0.69	NONE	-
C	EV	18	Reacts emotionally or aggressively to be touched	b1304 b1564 b2703	0.93 1 0.69	d2502	0
C	SE	19	Gets nervous (standing) near other people	b1520	0.91	d2501	0.49
C	SE	20	Rubs or scratches the spot where someone touches him/her	b1564 b755	1 0.39	NONE	-
C	BU	21	Touches people and objects without detecting social cues	b1304 b265	0.93 0.85	d1201 d2502	0.66 0
C	BU	22	Uncontrolled impulse for touch toys, surfaces or textures, intensely	b1304 b265	0.93 0.85	d1201	0.66
C	RE	23	(Lack of) awareness of pain	b2703 b280	0.69 1	NONE	-
C	RE	24	(Lack of) awareness of temperature changes	b2700	1	NONE	-
C	BU	25	Uncontrolled impulse for touching people and objects	b1304 b265	0.93 0.85	d2502	0
C	RE	26	Difficulties to notices when his/her face or hands are messy	b1564	1	NONE	-
D	BU	27	Intensive moving interferes with daily routines or requirements	b1470	0.84	d230	0.79
D	BU	28	Rocks back and forth repeatedly (chair/floor/standing up)	b1470 b7653	0.84 1	NONE	-
D	NQ	29	Scary emotions related to activities involving climb stairs	b1266 b1520	0.82 0.91	d4551	1
D	BU	30	(Enjoys) Positive feelings related to activities involving movement	b1301 b1520 b260	0.75 0.91 0.65	NONE	-
D	BU	31	Climbs or moves without concern for his/her own safety	NONE	-	d4551 d571	1 1
D	BU	32	Drops freely without concern for his/her own safety	b1144	0.66	d571	1
D	RE	33	Loss of balance walking in irregular surfaces	b2351 b7602	0.79 0	d4502	1
D	RE	34	Difficulties walking around obstacles	b7602	0	d4503	1
E	RE	35	Stiff movement pattern	b1470 b770	0.84 0	NONE	-
E	RE	36	Energy loss standing up or still	b1300	0.66	NONE	-
E	RE	37	Low tone in muscle groups	b735	0.66	NONE	-
E	RE	38	Looks for support to sustain himself, balance loss (hands; wall)	b2351 b755	0.79 0.39	d4154	0.66
E	RE	39	Requires foothold to keep balance	b2351	0.79	d4154	0.66
E	RE	40	Noisy gait pattern (feet noise)	b770	0	d450 d4556	1 0
E	BU	41	Stretches on people or furniture	b1470 b7600	0.84 0.66	NONE	-
E	NQ	42	Proprioceptive stimuli to sleep (heavy blanket)	b1564 b260 b2702	1 0.65 0.39	NONE	-
F	NQ	43	Hypersensitivity reactions (nausea) to food or textures	b1563 b250 b5350	0.23 0.93 0	d1203	0.93
F	SE	44	Hypersensitivity reactions to particular food flavors or scents (avoid)	b1562 b1563 b250	0 0.23 0.93	d1203	0.93
F	SE	45	Restricted his/her diet related to certain flavor	b1563 b250	0.23 0.93	d1203	0.93
F	SE	46	Restricted his/her diet related to certain texture	b1563 b250	0.23 0.93	d1203	0.93
F	SE	47	Restricted some food textures	b1563 b250 b265	0.23 0.93 0.85	d1203	0.93
F	BU	48	Smells things that are not food	b255	0.66	d1202	0
F	BU	49	Restricted dietary preferences related to some flavors	b1301 b1563 b250	0.75 0.23 0.93	d1203	0.93
F	BU	50	Special interest for some foods, flavors, or scents	b1301 b1563 b250	0.75 0.23 0.93	d1203	0.93
F	BU	51	Sensory preference for having things in the mouth	NONE	-	d1200	0
F	SE	52	Bites its tongue or lips frequently	b1470 b2703	0.84 0.69	NONE	-
G	RE	53	Difficulties related to detecting and avoiding risks	NONE	-	d571	1
G	RE	54	Paints, writes or draws hastily	b147 b1522	1 1	NONE	-
G	BU	55	Takes dangerous risks (low hazard awareness)	b1304	0.93	d571	1
G	BU	56	Intense needs for movement	b1300 b147	0.66 1	d2303	0
G	RE	57	Inefficient planning and executing tasks	b1641	1	NONE	-
G	EV	58	Stubborn or uncooperative	b1261	1	d2501	0.49
G	EV	59	Negative emotional response (throw tantrums)	b1253 b1521	0 0	d2503	0
G	BU	60	Pleasure emotions related to fall down	b1301 b1520 b260	0.75 0.91 0.65	NONE	-
G	EV	61	Avoids eye contact	b122	0	d1600	0.85
H	RE	62	Low self-esteem	b1265 b1266	0.66 0.82	NONE	-
H	EV	63	Needs external support to facing challenges	b1251 b1301	0.66 0.75	NONE	-
H	EV	64	Sensitivity to criticism	b1266	0.82	d7103	0
H	EV	65	Frequent defined and predictable fears	b1266 b1522	0.82 1	NONE	-
H	EV	66	Expresses failure feelings	b1265 b1266	0.66 0.82	NONE	-
H	EV	67	Overly serious	b1260 b1522	0.49 1	NONE	-
H	EV	68	Excessive emotional outburst when unable to finish a task	b1263 b1521	0 0	d2503	0
H	SE	69	Difficulties in comprehension of the literal and implied meaning of non-verbal messages	b16703	0	d3150	1
H	EV	70	Poor frustration tolerance	b1263 b1521	0 0	NONE	-
H	EV	71	Fears interfere in daily routine	b1266 b1522	0.82 1	d230	0.79
H	EV	72	Negative feelings related to changes on schedule plans, routine or expectations	b1250 b1521	1 0	d2304	1
H	SE	73	Needs more emotional or physical protection	b1266	0.82	d571	1
H	EV	74	Low participation or interaction in groups	b122 b1255 b1260	0 0 0.49	d7200	0
H	EV	75	Difficulty making/keeping friends	b122 b1260	0 0.49	d7500	0.66
I	RE	76	Eye contact lose during social interaction	b122	0	d1600 d7104	0.85 1
I	SE	77	Attention difficulties	b140	0.48	d160 d161	0.90 0.49
I	SE	78	Leaves a task uncompleted to attend to environmental features	b1400	0.85	d160 d210	0.90 1
I	RE	79	Seems indifferent in rich stimulation environments	b1264 b1400	1 0.85	d160	0.90
I	RE	80	Stares at objects	b140	0.48	d161	0.49
I	EV	81	Stares at people	b140	0.48	d1600 d161	0.85 0.49
I	BU	82	Stares at people movement	b1401	0	d1600 d161	0.85 0.49
I	BU	83	Difficulty focusing attention interfere in daily living activities	b1400	0.85	d160 d230	0.90 0.79
I	SE	84	Low orientation to place	b1141	1	NONE	-
I	RE	85	Negative emotions related to searching objects in complex environments	b140 b1520	0.48 0.91	NONE	-
I	RE	86	Difficulties in awareness of other individuals in one’s immediate environment.	b11421 b140	1 0.48	NONE	-

Section A - Auditory Processing; Section B - Visual Processing; Section C - Touch Processing; Section, Section D - Movement Processing; Section E- Body Position Processing; Section F- Oral Processing; Section G- Conduct associated with Sensory Processing; Section H- Socio-emotional response; Section I - Attentional response. The subtype of sensory response related to the item: Registration (RE); Seeking (BU); Sensitive (SE); Avoiding (EV); No Quadrant (NQ)

### Calculation of the Inter-Coder Agreement

The Kappa interrater agreement was computed for each ICF-CY code that was mapped for every meaningful unit (see Table 1). Cohen’s Kappa calculations for each ICF-CY code used revealed that the range of agreement varies between 0 (no agreement) to 1 (total agreement), depending on the code. Assuming every code was used only once, the mean Kappa value was 0.50 for the 86 items indicating a moderate level of agreement (Landis & Koch, 1977). When the mean Kappa is calculated considering every code and its frequency of use, the value increases to 0.68, indicating a substantial strength of agreement (Landis & Koch, 1977) possibly illustrating that both coders were consistent in the use of the codes.

The analysis of the PA between the two raters for each item showed that 80.23% of the SP-2 items were linked to the ICF-CY with total agreement, meaning that in an item both coders agree at least on one exact code (fourth level headings of the ICF-CY classification) for each item. This analysis shows a high level of agreement between the coders.

Furthermore, in 91.86% of cases, coders agreed on at least a third level of the ICF classification, in what was defined before as a partial agreement.

The coders only had a complete disagreement on the most precise code for summarizing the meaningful unit in 8.14% of the items.

### Diversity and Frequency of Codes

In the coding process, 98 different ICF-CY codes were used related to the 86 items of the SP-2 (see Table 2). Most of the SP-2 items were linked to body functions codes (61 of the 98 codes, 62.23%), no environmental factors codes were used (0%) and 37 codes related to Activities and participation were considered (37.77%).

**Table 2:** List of Codes used in the linking process and its international classification of functioning, disability and health description

CODE	ICF-CY DESCRIPTION	SP-2 ITEMS	TOTAL OF ITEMS
b11421	Orientation to others	86	1
b1144	Orientation to space	32	1
b122	Global psychosocial functions	61,74–76	4
b1250	Adaptability	72	1
b1251	Responsivity	63	1
b1253	Predictability	59	1
b1255	Approachability	74	1
b1261	Agreeableness	58	1
b1264	Openness to experience	79	1
b1265	Optimism	62,66	2
b1266	Confidence	29,62,64–66,71,73	7
b1300	Energy level	36,56	2
b1301	Motivation	9,10,30,49,50,60,63	7
b1304	Impulse control	18,21,22,25,55	5
b140	Attention functions	77,80,81,85,86	5
b1400	Sustaining attention	4,78,79,83	4
b1401	Sustaining attention	82	1
b147	Psychomotor functions	54,56	2
b1470	Psychomotor control	27,28,35,41,52	5
b1520	Appropriateness of emotion	1,8,11,13,16,17,19,29,30,60,86	11
b1521	Regulation of emotion	59,68,70,72	4
b1522	Range of emotion	54,65,67,71	4
b1560	Auditory perception	1–8	8
b1561	Visual perception	9–11,13–15	6
b1562	Olfactory perception	44	1
b1563	Gustatory perception	43–47,49,50	7
b1564	Tactile perception	16–18,20,26,42	6
b1565	Visuospatial perception	12	1
b1641	Organization and planning	57	1
b16703	Reception of gestural language	69	1
b21020	Light sensitivity	9,13,15	3
b2351	Vestibular function of balance	33,38,39	3
b250	Taste function	43–47,49,50	7
b255	Smell function	48	1
b260	Proprioceptive function	30,42,60	3
b265	Touch function	21,22,25,47	4
b2700	Sensitivity to temperature	24	1
b2702	Sensitivity to pressure	16,17,42	3
b2703	Sensitivity to a noxious stimulus	1,2,13,15–18,23,52	9
b280	Sensation of pain	23	1
b3401	Making a range of sounds	8	1
b5350	Sensation of nausea	43	1
b735	Muscle tone functions	37	1
b755	Involuntary movement reaction functions	2,20,38	3
b7600	Control of simple voluntary movements	41	1
b7602	Coordination of voluntary movements	33,34	2
b7653	Stereotypies and motor perseveration	28	1
b770	Gait pattern functions	35,40	2
d110	Watching	14	1
d1200	Mouthing	51	1
d1201	Touching	21,22	2
d1202	Smelling	48	1
d1203	Tasting	43–47,49,50	7
d160	Focusing attention	4,7,77–79,83	6
d1600	Focusing attention on the human touch, face and voice	6,61,76,81,82	5
d1601	Focusing attention to changes in the environment	12	1
d161	Directing attention	77,80–82	4
d210	Undertaking a single task	3,5,78	3
d230	Carrying out daily routine	27,71,83	3
d2303	Managing one’s own activity level	56	1
d2304	Managing changes in daily routine	72	1
d2501	Responding to demands	19,58	2
d2502	Approaching persons or situations	18,21,25	3
d2503	Acting predictably	59,68	2
d3100	Responding to the human voice	6,7	2
d3150	Communicating with - receiving - body gestures	69	1
d4154	Maintaining a standing position	38,39	2
d450	Walking	40	1
d4502	Walking on different surfaces	33	1
d4503	Walking around obstacles	34	1
d4551	Climbing	29,31	2
d4556	Shuffling	40	1
d571	Looking after one’s safety	31,32,53,55,73	5
d7103	Criticism in relationships	64	1
d7104	Social cues in relationships	76	1
d7200	Forming relationships	74	1
d7500	Informal relationships with friends	75	1

Given the possibility of linking multiple codes for the same meaningful unit, it was also analyzed the frequency of use of the codes. The complete linking process resulted in a sum of 218 codes (see Table 1). Out of those, 155 (71%) were codes related to the body functions chapter, mainly linked to perceptual functions (b160), emotional functions (b 152) and temperament and personality functions (b126) of the ICF-CY. Concerning the activities and participation chapters, codes referred to those ICF-CY chapters were used 63 times (29%). The most frequently used codes of the activities and participation chapters were focusing attention (d160), carrying out a daily routine (d230) and walking (d450).

### Proportion Covered from the Autism Core Set by SP-2

ASD ICF-CS (Bölte et al., 2019; Schiariti et al., 2018) is composed of 111 ICF categories (all at the 2nd level): 1 related to body structures, 20 to body functions, 59 to activities and participation and 31 to environmental factors. As is defined in our linking process, the SP-2 covers 70% of the ICF categories from the ASD ICF-CS related to body functions (14 of the 20 codes) and 20% of the components referred to activities and participation (12 of the 59 codes).

SP-2 covers 23.42% of the categories described in the ASD ICF-CS (26 out of the 111 components), 12.61% of the codes refer to body functions, and 10.8% of them refer to activities and participation (12 out of 111). No codes were related to environmental factors.

**Fig. 3:**
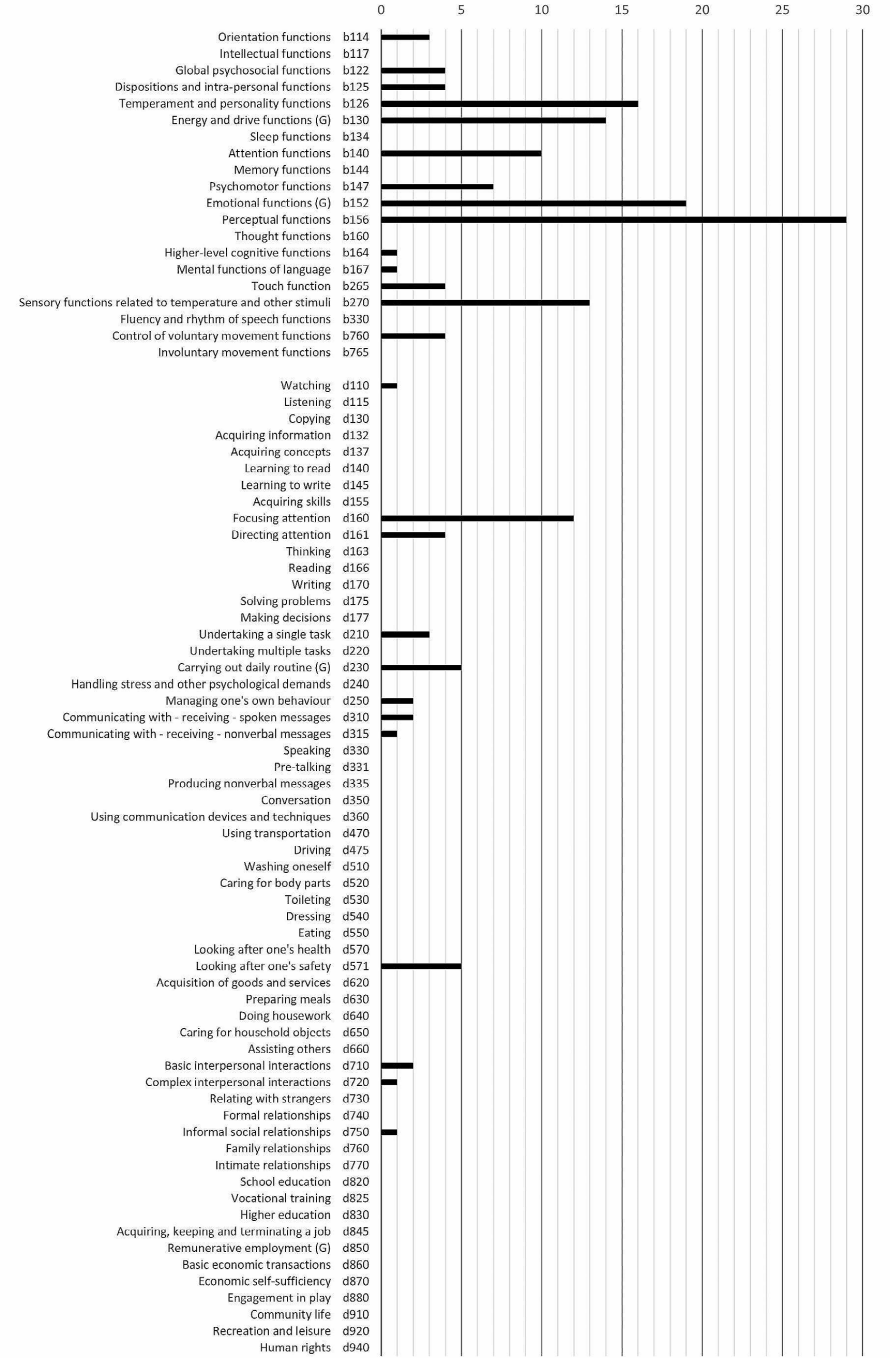
Distribution of the resulting codes of the SP-2 linking process related to the International Classification of Functioning, Disability and Health Core Set for Autism. Axis X: ICF Code name, ICF code number; Axis Y: frequency of use along the Sensory Profile-2 (SP-2)

Coverage of the ASD ICF-CS range with the SP-2 is represented in Fig. 3. The frequency of use of every code in the final linking process is also indicated in Fig. 3 to illustrate the results described before.

## Discussion

The utility of the ICF framework for analyzing the functioning facets of health assessment tools is well documented. This knowledge is crucial for clarifying the questions of what and how to measure and for guiding clinicians in selecting appropriate instruments (Boldt et al., 2005). Therefore, this study aims to contribute to enriching this field of knowledge by examining one of the most commonly used scales in autism: SP-2. This instrument focuses on sensory dimensions, which is, among other, part of the diagnosis criteria for ASD and thus need to be assessed, in tandem with other dimensions, to understand children with ASD functionality. The construct analysis of the SP-2 contents with the ICF-CY made it possible to detect and quantify the concepts related to every item of the scale from the ICF framework and also highlighted and clarified the structure and utility of the scale.

Firstly, the study of the agreement level between ICF-CY trained researchers when assigning content to the ICF-CY functioning dimensions was consistent with previous studies that linked measurement instrument items with the ICF-CY classification system. The mean kappa values of these studies ranged from 0.22 to 0.76 (Black et al., 2023; Castro et al., 2013; Castro et al., 2018; Dahlgren et al., 2013; Sogo, 2020). Regarding our results, it is important to note that Cohen’s Kappa coefficient has an average score from 0.50 to 0.68, indicating a high level of agreement between the two principal coders during the linking process. Mainly, this statistical approach confirms that the resulting codes did not come about by chance. This feature aligns with the high percentage of agreement (PA) that oscillates between 80.23% and 91.86% of agreement. The use of the PA analysis in the Linking process is less common, but it can be helpful in determining the degree of agreement for the code and the item indistinctly.

Secondly, our results show the distribution of the SP-2 codes across the ICF-CY chapters and reveal that all the concepts represented in the SP-2 items could be assigned to ICF-CY codes following the linking rules. Most of these codes were related to body function chapters and show that the SP-2 is particularly helpful in assessing perceptual functions (b156), emotional functions (b152) and temperament and personality functions (b126).

Thirdly, the identification of the functional dimensions assessed by SP-2, regarded as essential from the ASD Core Set perspective, shows that, even though the SP-2 items do not assess the overall majority of the functioning features, the SP-2 encompasses a majority of aspects concerning body functions. Therefore, this instrument should be considered as part of a multidimensional assessment that includes other tools capable of assessing activities, participation, or environmental factors. Previous works show that several measures typically used for the assessment of ASD heavily focus on activities and participation areas and they are not comprehensive enough to cover the three domains of the ICF (D’Arcy et al., 2022). Specifically, in what is related to environmental factors, literature reveals that these are often neglected by the most used assessment tools for ASD (Castro et al., 2013; Black et al., 2023; D’Arcy et al.,^2022^; Hayden-Evans et al., 2022; Sogo, 2020). To provide an accurate and holistic picture of an individual’s abilities and support needs, it is necessary to assess the presence of specific environmental supports and barriers and how they interact with the individual’s activities and participation (WHO, 2001).

Some limitations of this study should also be pointed out. Analyzing the assessment tool and developing the meaningful units required a deep understanding of Dunn’s Sensory Processing Model (1997). Therefore, preliminary training was needed for the three coders and lengthened the start of the process. Also, this highlights the requirement of adequate training for every clinician who wants to use this tool as part of their assessment process.

Future research on this topic should keep two distinct approaches: On the one hand, researchers should continue developing the mapping process between the most used measurement tools and the ICF framework, as well as link the elements of the ICF-CS with existing measurement tools. It should be noted that future ICF linking processes with ASD assessment tools, should consider the deliberations addressed in the revised ASD ICF-CS by Bölte et al., 2024. Such mapping would provide researchers and clinicians with a useful road map to identify the most suitable instruments to evaluate all the essential functioning dimensions.

On the other hand, as Wright (2015) pointed out, ICF-CS highlights what is important to be measured in different health conditions, but does not address how to measure those areas of functioning. Because of that, the development of ICF-based instruments is crucial to ensure a functional assessing-intervention process. ICF-CS can serve as an item pool for developing assessment instruments (Mahdi et al., 2018, Selb, 2015) and guide the researchers in the developing and validating process of new instruments (Bölte et al., 2019). These kinds of measures, resulting from the ICF framework, can improve their clinical utility as they promote more holistic, biopsychosocial, and functional interventions (Hayden-Evans et al., 2022).

To the best of our knowledge, the current study is the only one to conduct a construct content analysis of a sensory processing measure using the ICF-CY framework, and specifically the first to analyze the Sensory Profile™ 2 (Dunn, 2014) from an ICF perspective. Therefore, this study is original and may stimulate future research on this scale. Additionally, it may lead to the development of a proposal for an ICF Core Set-based toolbox of measures for ASD, providing comprehensive coverage of the ICF codes included in the ASD ICF-CS, as it has been developed for other health conditions such as cerebral palsy (Schiariti et al., 2017). Furthermore, this study distinguishes itself from previous ones by including clear and replicable methodological procedures, as well as additional statistical analyses (such as the percentage of agreement), which enhance the robustness of the results.

To conclude, the ICF-CY is a valuable reference to identify and quantify the concepts in the SP-2. Furthermore, a comparison between the SP-2 and the ICF-CS has provided insights into the areas covered by this instrument and highlights the need for a multidimensional assessment in ASD that allows for the recognition of the different dimensions of symptom expression that are critical to the success of care planning. The reliable assessment of functioning in children with ASD is increasingly becoming a priority, given the daily impairments borne by individuals with this disorder and the influence that assessment has on the resulting intervention program.
